# Update on Phytochemistry and Pharmacology of Naturally Occurring Resveratrol Oligomers

**DOI:** 10.3390/molecules22122050

**Published:** 2017-11-24

**Authors:** Jie Shen, Qiang Zhou, Pei Li, Zhiqiang Wang, Shuangshuang Liu, Chunnian He, Chunhong Zhang, Peigen Xiao

**Affiliations:** 1 Institute of Medicinal Plant Development, Chinese Academy of Medical Science, Peking Union Medical College, Beijing 100193, China; jieshen0817@163.com (J.S.); zhouqiang1030@126.com (Q.Z.); Lee_p1214@163.com (P.L.); wzqwillis1016@gmail.com (Z.W.); ben_jiu_ru_ci@163.com (S.L.); pgxiao@implad.ac.cn (P.X.); 2 Laboratory of Bioactive Substances and Resources Utilization of Chinese Herbal Medicine, Ministry of Education, Beijing 100193, China; 3 School of Graduate, Tianjin University of Traditional Chinese Medicine, Tianjin 300193, China; 4 School of Pharmacy, Anhui Medical University, Hefei 230032, China; 5 School of Pharmacy, Baotou Medical College, Baotou 014060, China

**Keywords:** resveratrol oligomers, distribution, phytochemistry, pharmacology

## Abstract

Resveratrol oligomers (REVs), a major class of stilbenoids, are biosynthesized by regioselective oxidative coupling of two to eight units of resveratrol monomer. Due to their unique structures and pleiotropic biological activities, natural product chemists are increasingly focusing on REVs in the last few decades. This study presents a detailed and thorough examination of REVs, including chemical structures, natural resources, and biological activities, during the period of 2010–2017. Ninety-two new REVs compounds, including 39 dimers, 23 trimers, 13 tetramers, six resveratrol monomers, six hexamers, four pentamers, and one octamer, have been reported from the families of Dipterocarpaceae, Paeoniaceae, Vitaceae, Leguminosae, Gnetaceae, Cyperaceae, Polygonaceae Gramineae, and Poaceae. Amongst these families, Dipterocarpaceae, with 50 REVs, accounts for the majority, and seven genera of Dipterocarpaceae are involved, including *Vatica*, *Vateria*, *Shorea*, *Hopea*, *Neobalanocarpus*, *Dipterocarpus*, and *Dryobalanops*. These REVs have shown a wide range of bioactivities. Pharmacological studies have mainly focused on potential efficacy on tumors, bacteria, Alzheimer’s disease, cardiovascular diseases, and others. The information updated in this review might assist further research and development of novel REVs as potential therapeutic agents.

## 1. Introduction

Resveratrol oligomers (REVs), a major class of stilbenoids, are commonly biosynthesized by regioselective oxidative coupling of two to eight units of resveratrol monomer [1]. Some plants containing REVs have been used for a long time in traditional Asian medicine. For example, *Nardostachys chinensis* Batal (Valerianaceae), a traditional herbal tranquilizer in China, contains REVs as the active ingredient. The resin of *Shorea* species, rich in REVs, has been used as an astringent to treat diarrhea and dysentery in Malaysia [2,3,4,5]. Several pure REVs have been isolated from various plants. Based on pharmacological studies, REVs are reported to have multi-faceted biological activities [6,7,8], including antimicrobial, antioxidant, and antitumor effects, as well as cardiovascular protection. Due to their unique chemical structures and diverse biological activities, REVs have increasingly captured the attention of medicinal chemists [9,10].

As the research information on REVs has accumulated, several reviews appeared from 2010 to 2017 [1,11,12,13,14,15,16,17,18,19]. In 2013, the biosynthesis, chemistry, and proprieties of REVs in grapes were summarized by Riccardo et al. [11]; the structures of oligostilbenoids in Dipterocarpaceae plants and their biological activities were reviewed [12]. Another review published in 2013 [13] summarized 60 stilbenes, including 27 REVs found from 2009 to 2013, but the source plants were not mentioned. In 2014, Lim et al. reviewed resveratrol and its oligomers in modulating sphingolipid metabolism and signaling in diseases [14]; another article in 2014 reviewed REVs for the prevention and treatment of cancers [15]. Of two papers published in 2015, one focused on biosynthesis as well as some bioactivities [16]; another one focused on the diverse bioactivities of ologostibenes [17]. One paper in 2017 [18] chose “cancer chemopreventive potential” as the topic.

As phytochemistry and pharmacology of naturally occurring REVs has progressed, our review provides a detailed and thorough examination of their chemical structures, natural resources, and biological activities, from 2010 to 2017. In this period, 92 new REVs were isolated and identified, including 39 dimers, 23 trimers, 13 tetramers, six resveratrol monomers, six hexamers, four pentamers, and one octamer. However, no resveratrol heptamer has been discovered. These REVs are mostly distributed in the following seven families: Dipterocarpaceae, Paeoniaceae, Vitaceae, Leguminosae, Gnetaceae, Cyperaceae, and Polygonaceae. Most REVs were isolated from the family of Dipterocarpaceae, as in the past. The pharmacological activities of REVs are mainly concentrated on antibacterial, antioxidant, anti-Alzheimer’s disease, anti-Parkinson’s disease, anti-tumors, and cardiovascular protection, as well as liver protective effects. The review aims to provide readers with comprehensive information on the progress of REVs.

## 2. Phytochemistry

**Figure 1:**
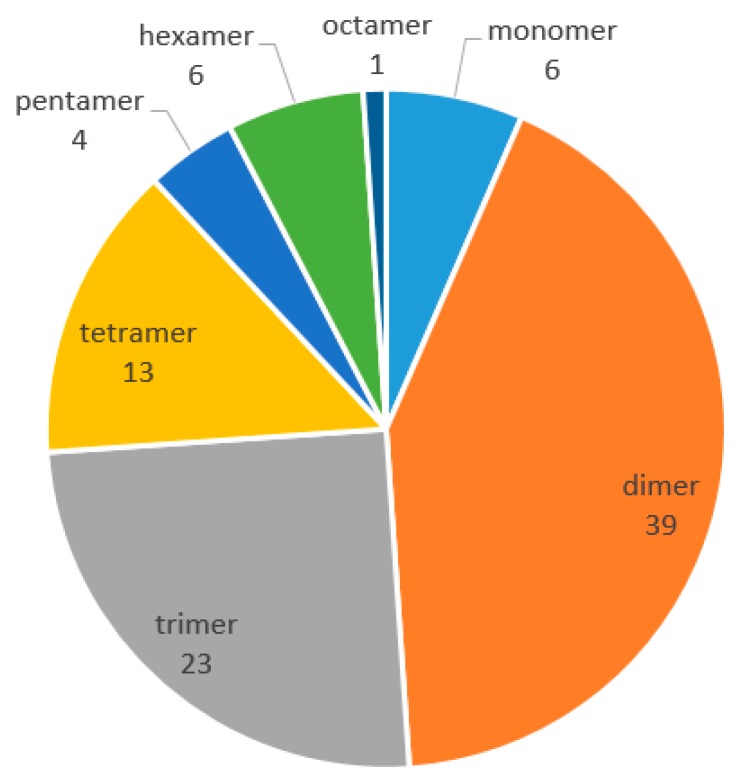
The polymerization situations of resveratrol oligomers from 2010 to 2017.

**Figure 2:**
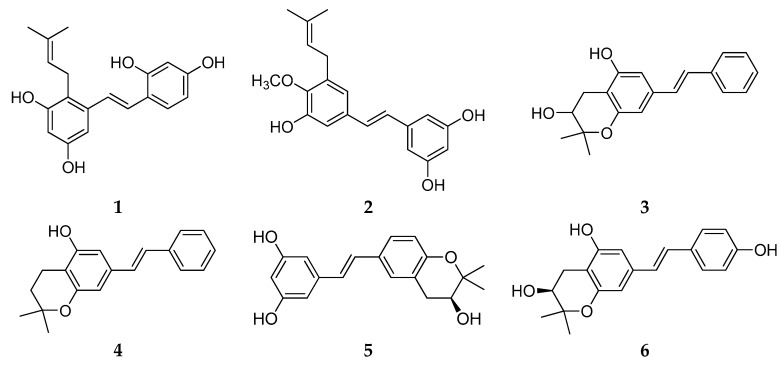
The structure of resveratrol monomers.

**Figure 3:**
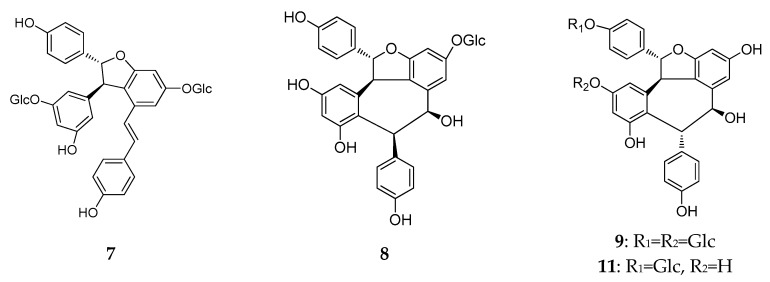

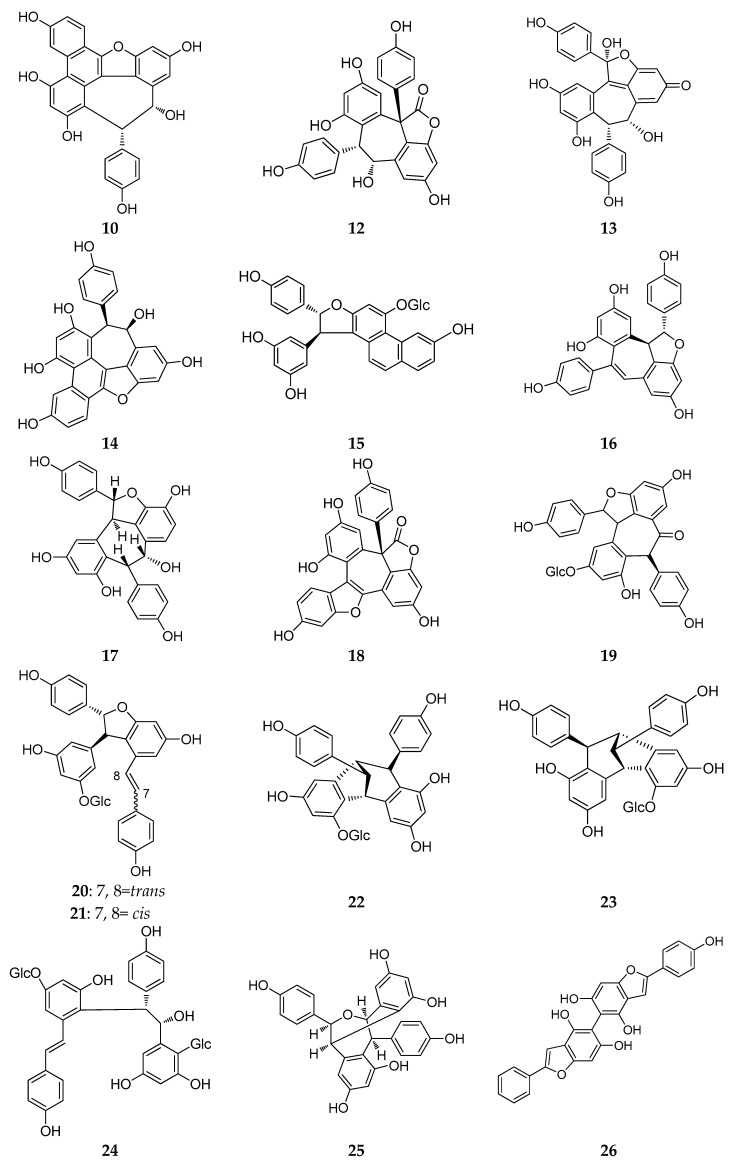

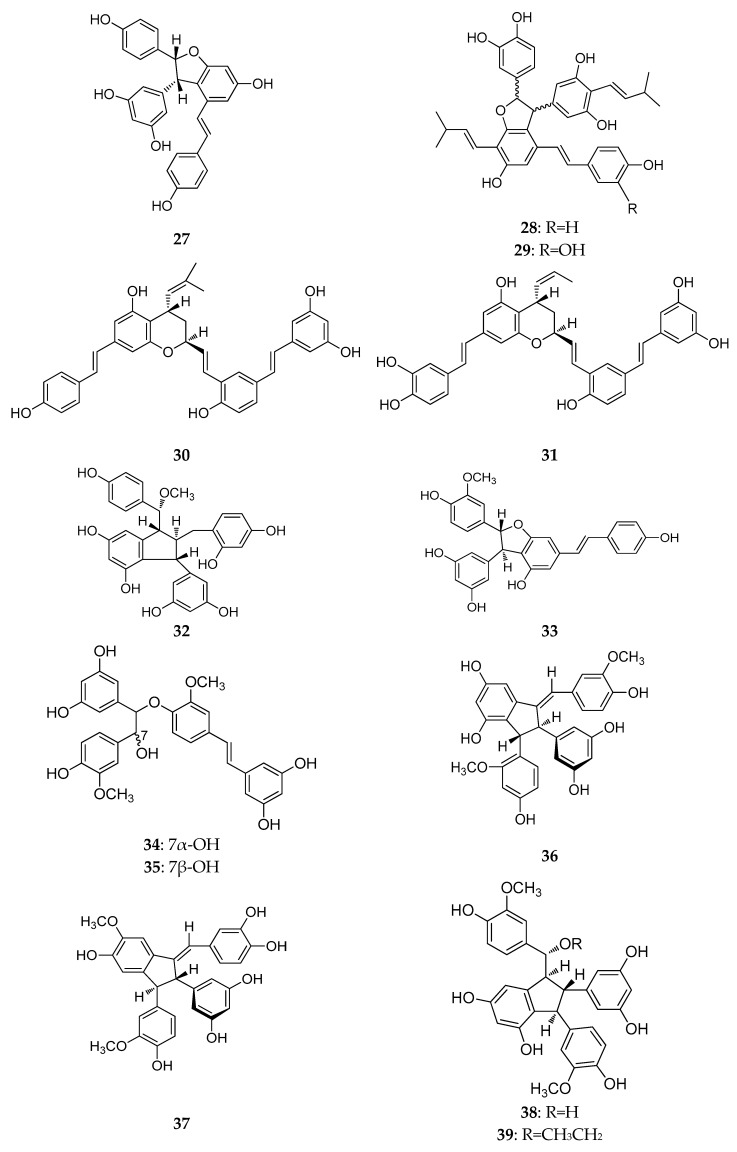

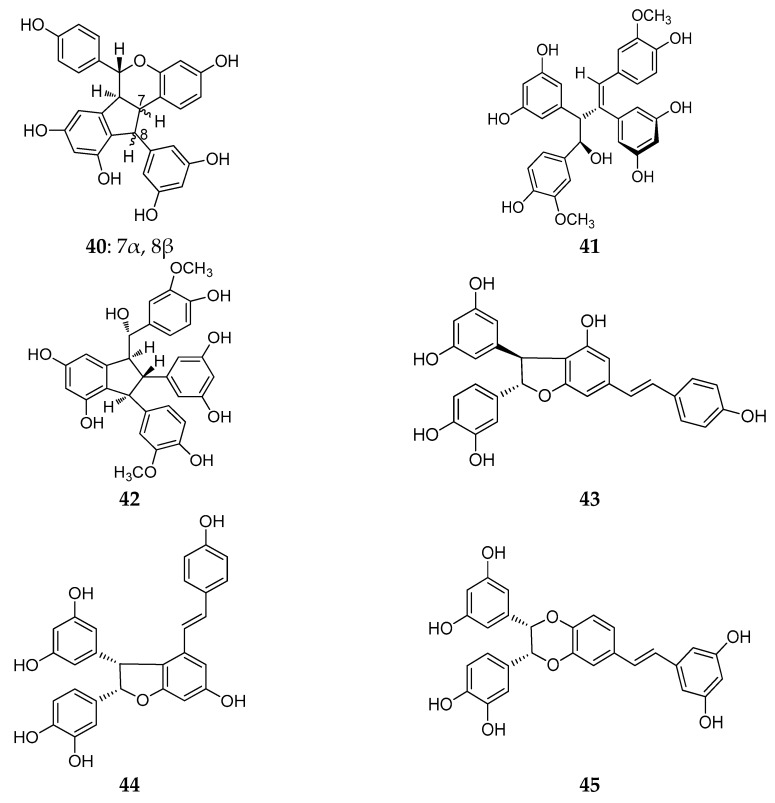
The structures of resveratrol dimers (**7**–**45**).

**Figure 4:**
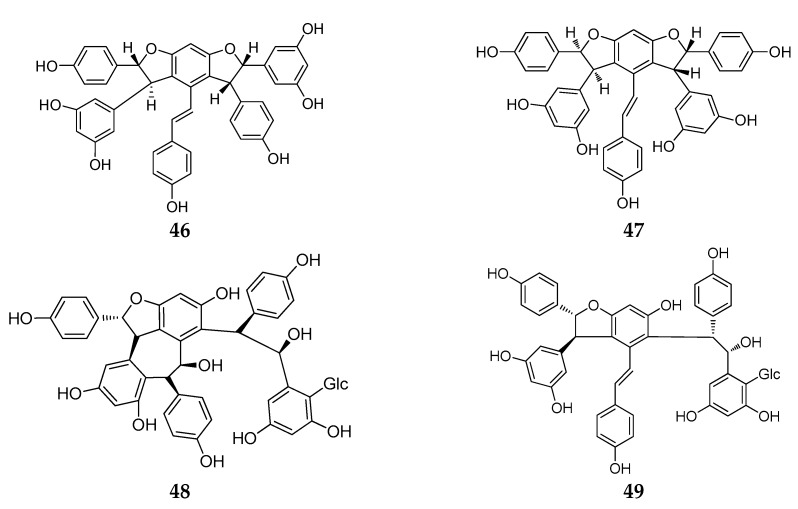

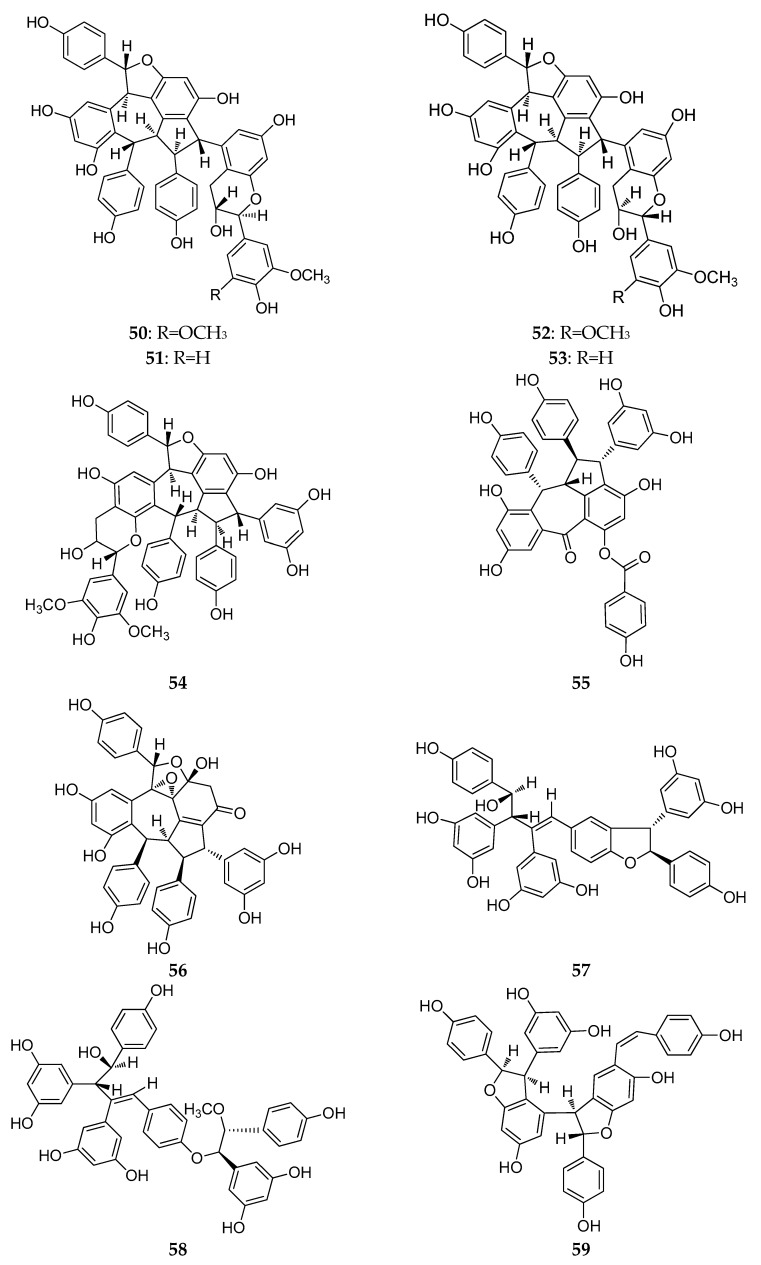

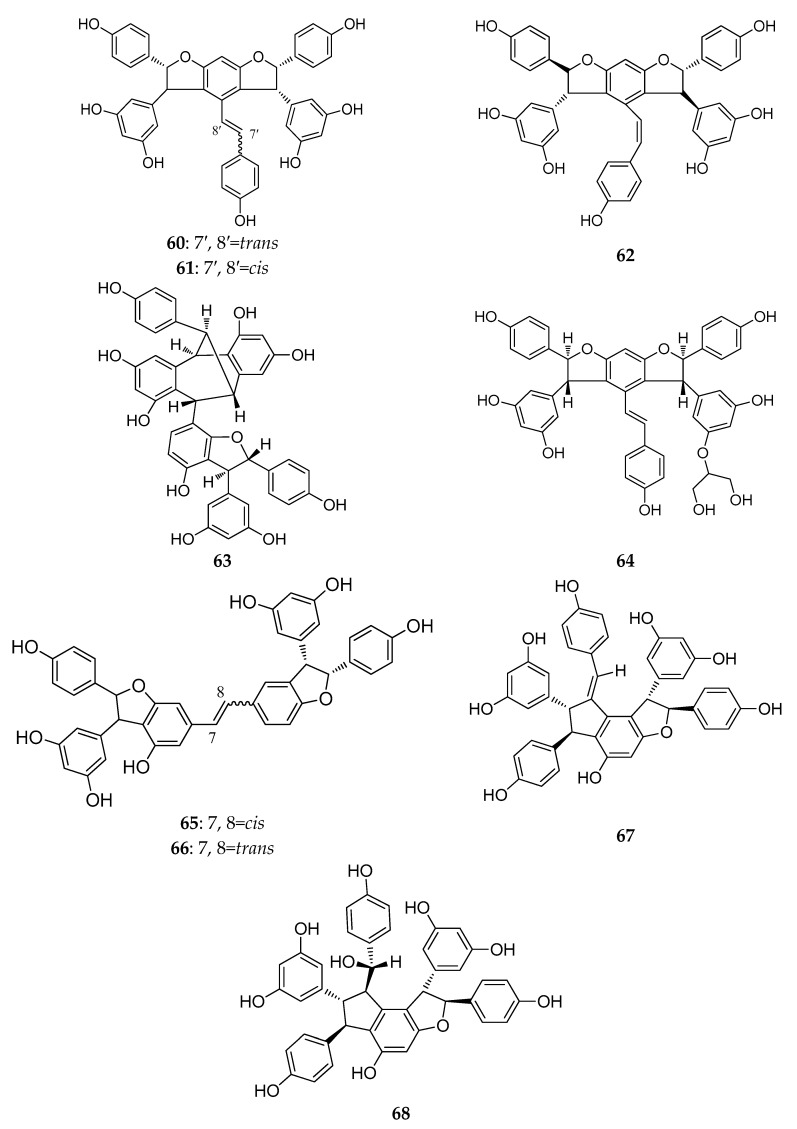
The structures of resveratrol trimers (**46**–**68**).

**Figure 5:**
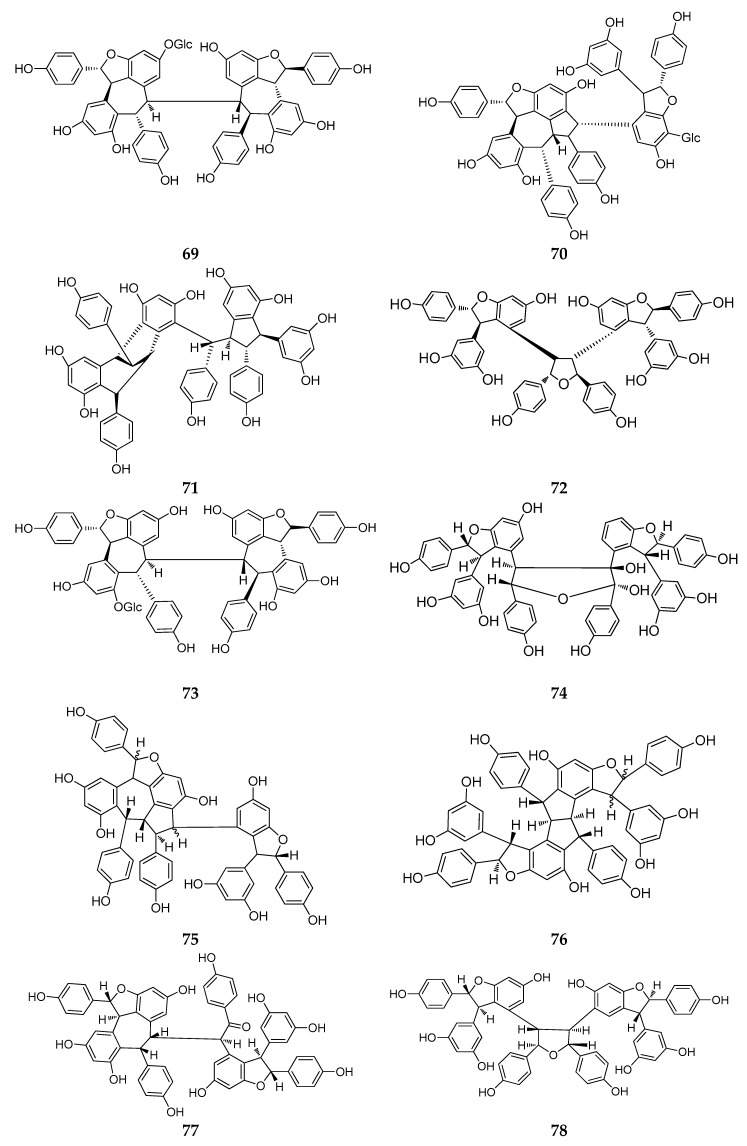

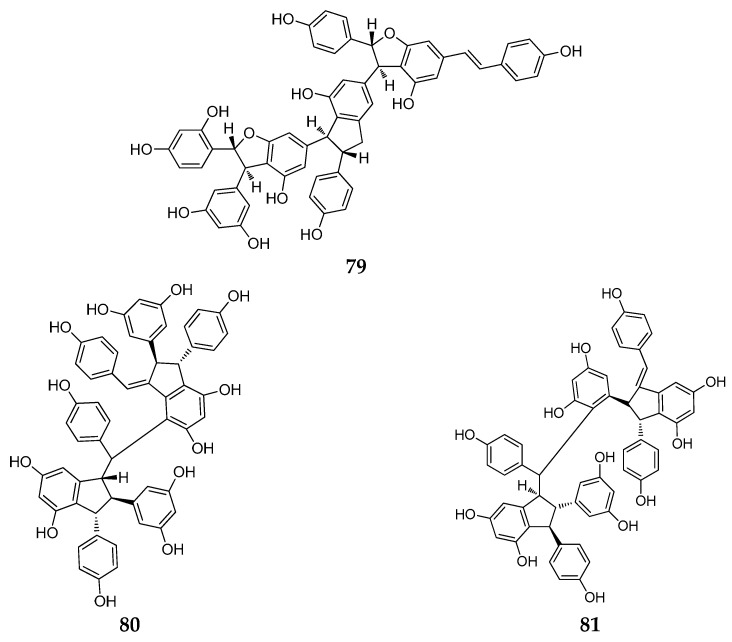
The structures of resveratrol tetramers (**69**–**81**).

**Figure 6:**
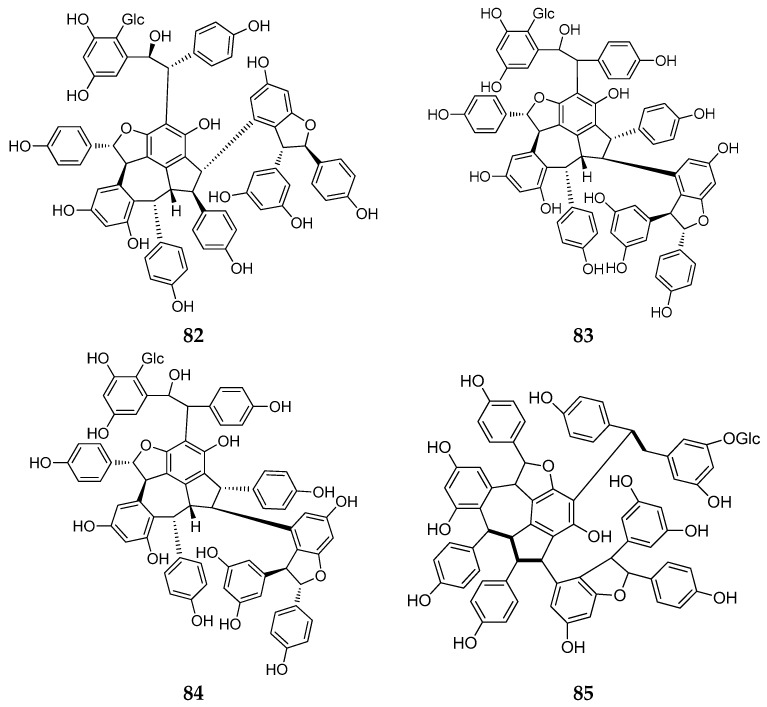
The structures of resveratrol pentamers (**82**–**85**).

**Figure 7:**
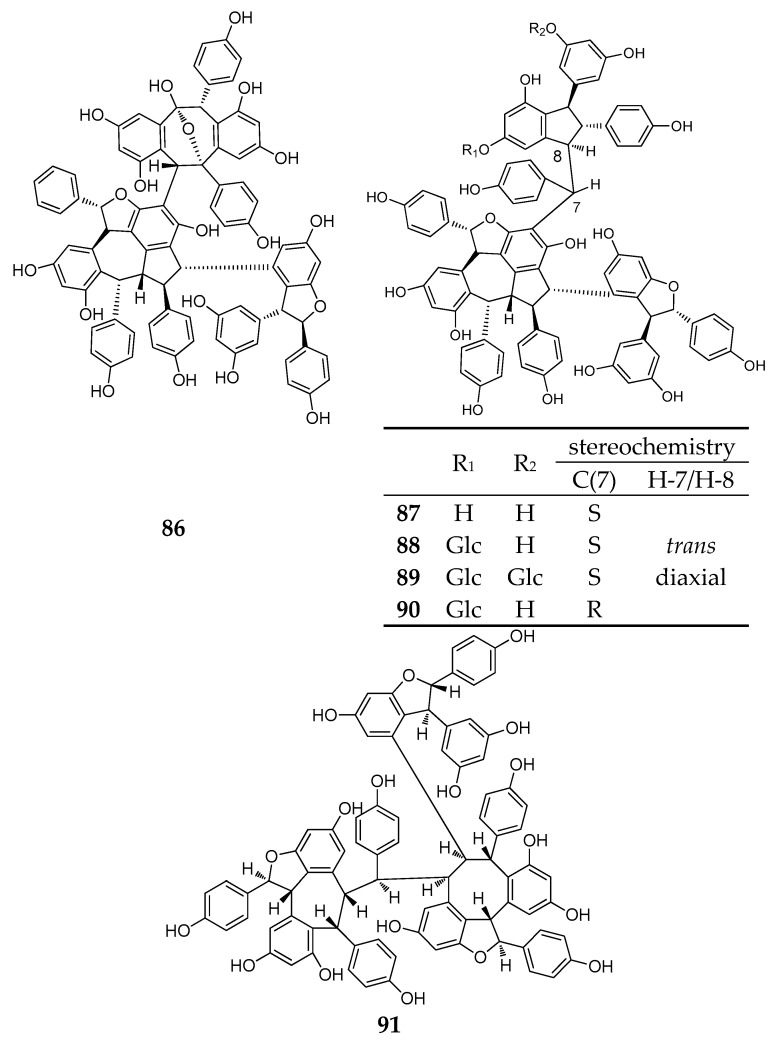
The structure of resveratrol hexamers (**86**–**91**).

**Figure 8:**
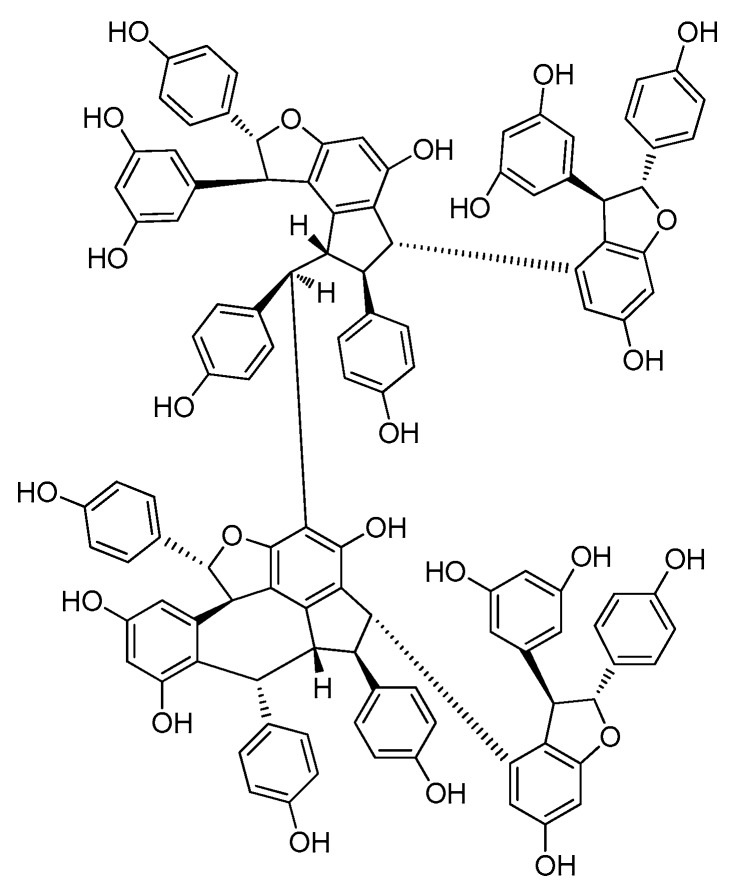
The structure of resveratrol octamer (**92**).

Chemical structure analysis showed that REVs were polymerized from two to eight, or even more, resveratrol units that have great structural diversity and include, but are not limited to, the following features: (1) a degree of polymerization up to eight; (2) formation of dihydrobenzofuran(s) or a benzofuran or a chroman; (3) O-glucosylation; (4) condensation of a phenylpropanoid; and (5) formation of a cyclohexa-2,5-dienone. Chemical structures of the 92 new REVs from dimer to octamer are compiled in Figure 1, Figure 2, Figure 3, Figure 4, Figure 5, Figure 6, Figure 7 and Figure 8, and their plant sources are listed in Table A1 in Appendix A.

### 2.1. Resveratrol Monomers

Resveratrol monomers are compounds that possess one stilbene skeleton with various substituting groups. Many resveratrol derivatives have been found in natural products and have been obtained from chemical synthesis and structure modifications. Due to the simple structures and diverse biological activities, resveratrol monomers have been intensively studied. Six new resveratrol monomers were successfully isolated from 2010 to 2017, though the speed of discovery has decreased (Table A1; Figure 2).

Shan et al. found a new prenylated resveratrol derivative, cudrastilbene (**1**), from the roots of *Cudrania tricuspidata* [19]. A new resveratrol derivative (**2**) (3,5,3′-trihydroxy-4′-methoxy-5′-isopentenylstilbene, MIP) was isolated from black skin peanut seeds that had been attacked by the fungal strain *Rhizopus oligoporus* [20]. Three new resveratrol derivatives were successfully isolated from peanut seeds infected by an *Aspergillus flavus* strain, along with chiricanine B (**3**). Chiricanine B was not previously reported in peanuts, but was reported as a synthetic stilbenoid product. The structures of three new putative phytoalexins were named as arahypin-13 (**4**), arahypin-14 (**5**), and arahypin-15 (**6**) [21]. Resveratrol derivatives (**1**, **4**, **5** and **6**) were all produced in the infected seeds. These new compounds might have a role in defense against invasive fungi.

### 2.2. Resveratrol Dimers

A total of 39 resveratrol dimers (**7**–**45**) were isolated and distributed in six plant families: Dipterocarpaceae, Vitaceae, Paeoniaceae, Leguminosae, Gnetaceae, and Cyperaceae. (Table A1, Figure 3).

In this period, more than half (21/39) of the resveratrol dimers (**7**–**22**) were discovered in leaves, stems, barks, and the heart wood of Dipterocarpaceae plants, and 10 have the moiety of benzofuran (**7**–**11**, **13**–**17** and **23**–**25**) [22,23,24,25,26,27,28,29]. Additionally, vatalbinosides C (**7**) and E (**9**) are the first Dipterocarpaceae that possess two *O*-d-glucopyranosyl moieties [22,23]. The planar structure of vaticahainols B (**13**) has an unusual quinone ring, and vaticahainols C (**14**) has a unique phenanthrene group [24]. Roxburghiol A (**16**) has the same absolute configurations as (−)-*ε*-viniferin [26]. Upunosides F (**20**) and G (**21**) are two dimeric aglycones [29]. Another dimer, vaticahainol A (**12**), shows rearrangement from the original resveratrol unit and contains a lactone moiety [24]. The planar structure of dipterocarpols A (**18**), a rearranged resveratrol dimer of hopeahainol A, also contains a lactone moiety, and dipterocarpols B (**19**) was determined as hopeafuran-*O*-*β*-glucopyranoside [28]. Two new isomeric *O*-glucosides of resveratrol dimers, ampelopsin F-11b-*O*-*β*-glucopyranosides, with the enantiomeric aglycones cordifoloside A (**22**), and cordifoloside B (**23**), are the first REVs that demonstrate the co-occurrence of diastereomeric *O*-glucosides with enantiomeric aglycones in this family [30]. Hopeasides D (**24**) is a new resveratrol dimer *C*-Glucoside, possessing a novel substituent, the 1-hydroxy-1-(3,5-dihydroxy-2-*C*-glucopyranosylphenyl)-2-(4-hydroxyphenyl) ethane-2-yl group [31]. Heimiol B (**25**) is a tricuspidatol A derivative that has two additional symmetrically attached resveratrol units [32].

In China, plants from the *Vitis* genus (Vitaceae) have been used in traditional medicines. The roots and stems of *Vitis amurensis* Rupr. can alleviate pain from injury, rheumatalgia, stomachache, neuralgia, and others. Yao et al. found several REVs in *Vitis amurensis*, and a new resveratrol dimer, amurensin O (**26**), obtained from the roots of *Vitis amurensis.* Amurensin O is connected by two benzofuran stilbene monomers through a C–C bond [33].

During this period, only one new resveratrol dimer, named (−)-7a,8a-*cis*-*ε*-Viniferin (**27**), was isolated from the seeds of *Paeonia lactiflora* (Paeoniaceae). A compound (**27**) that was synthesized from resveratrol, by FeCl_3_ treatment, was isolated from natural resources [34].

When infected by fungi, the peanut can produce a unique series of REVs to protect themselves. *Aspergillus* species were used to infect some types of peanut seeds to obtain stilbene phytoalexins. Sobolev et al. isolated two new prenylated resveratrol dimers, named arahypin-6 (**28**) and arahypin-7 (**29**), from peanut seeds that were wounded by the fungal strain *Aspergillus caelatus* [35]. Liu et al. used *Rhizopus oligoporus* to infect black skin peanut seeds and discovered two new resveratrol dimers, arahypin-11 (**30**) and arahypin-12 (**31**) [20].

The plants of genus *Gnetum* (Gnetaceae), widely recognized as abundant sources of REVs, are mainly found in northeastern Thailand. Sri et al. isolated two new REVs, macrostachyols C and D (**32**, **33**) from the roots of *Gnetum macrostachyum* [36].

Twenty-seven stilbenoids, including 24 stilbene dimers, are polymerized in many ways, such as one bond of 8-*O*-4′ (**34** and **35**) or 8-8′ (**41**), two bonds forming an indane (**36**–**39**), or a benzofuran, and three bonds forming an indano[1,2-*c*]-chromene (40), a dibenzobicyclo [3.2.1] octane, an indano[2,1-*a*] indane, or a benzo [6,7] cyclohepta [1,2,3-*cd*] benzofuran, were isolated from the caulis of *Gnetum macrostachyum*. Among them, gnemontanins A-G (**34**–**40**) as well as (−)-gnetuhainin P (**41**) and (−)-gnetuhainin I (**42**) were structurally determined as new compounds. Naturally occurring stilbene dimers, polymerized through one bond of 8-*O*-4′ (**34** and **35**), as well as two bonds of 7–8′ and 6–7′ (**37**) were reported for the first time [37]. 

Through bioassay-guided separation, three new resveratrol dimers, longusols A–C (**43**–**45**) were isolated from the *Cyperus longus* [38]. Longusol A (**43**) showed a similar carbon skeleton as longusol B (**44**), which is connected with two resveratrol monomers by a common benzofuran ring. In addition, longusol B (**44**) exhibited the opposing stereostructure, similar to the *cis*-type isomer in the dihydrofuran part of *trans*-scirpusins A. Longusol C (**45**), composed of two resveratrol units connected by a 1,4-dioxane moiety, and the stereoisomer was determined to be *cis*-type isomer in 1,4-dioxane part of cassigarols E [38].

### 2.3. Resveratrol Trimers

Resveratrol trimers are composed of three resveratrol units and connected by a head-to-ligation or circular structure [12]. Similar to the resveratrol dimer, 23 resveratrol trimers (**46**–**68**) have been obtained since 2010 from five plant families: Dipterocarpaceae, Vitaceae, Paeoniaceae, Gnetaceae and Polygonaceae. Dipterocarpaceaeis is the main source of resveratrol trimers, and 11 resveratrol trimers (**46**–**56**) were isolated from this family during this period (Table A1; Figure 4).

*Dryobalanops* is a unique genus in the Dipterocarpaceae family that only lives in the tropical forests of west Malysia with only seven species worldwide [39]. Two trimers, malaysianol A (**46**) and malaysianol D (**47**), were isolated from *D. aromatic* and *D. beccarii*, respectively [40,41]. Interestingly, malaysianol A (**46**) has a unique biogenetic pathway, arranged with a dimer of *ε*-viniferin and one free resveratrol monomer from oxidative coupling. Malaysianol D (**47**) is a symmetrical trimer [41]. The *Hopea genus* is known for richness of biologically active REVs. The phytochemical investigation of the stem of *H. utilis* resulted in a new resveratrol trimer: hopeaside E (**48**), which is composed of three resveratrol units through oxidative condensation, and is the first instance of a *C*-glucoside of a resveratrol oligomer possessing two aliphatic OH groups in aglycone [42]. Hopeasides C (**49**) is a resveratrol trimer possessing the same novel substituent as the dimer of hopeasides D (**20**) [29]. Cheng and co-workers completed a phytochemical investigation of the stem bark of *H. chinensis* and isolated five new resveratrol trimers, hopeachinols E–I (**50**–**54**) [43]. All these trimers possess a novel REV carbon skeleton in which a resveratrol trimer is associated with one lignan monomer via a pyran ring. The biosynthetic origin of these trimers is associated with the same resveratrol trimer, vaticanol A, through continuing the cyclization reaction of the intramolecular free radical with two-carbon units or phenylpropanoid [43]. The final two trimers, dipterocarpols C (**55**) and D (**56**) were identified from the stem wood of *Dipterocarpus alatus* [31]. Notably, the discovery of dipterocarpols C (**55**) was the first case where the biosynthetic origin of resveratrol aneuploids was correlated with the loss of a half resveratrol unit through oxidative cleavage [31].

Gu and coworkers reported the isolation of a trimer, wenchowenol (**57**), from the roots and stems of *Vitis wenchowensis*, and concluded that the biosynthetic origin of wenchowenol (**57**) was linked to amurensin A and resveratrol by oxidative coupling [44]. Another new trimer, quinquangularol (**58**), contains a similar biosynthetic pathway to wenchowenol (**57**); one difference is a methylation step followed by the oxidative coupling between amurensin A and resveratrol [45]. From the grapevine shoot extracts of *Vitis vinifera*, a novel resveratrol trimer, (*Z*)-*cis*-miyabenol C (**59**), was isolated, which possesses a *cis*-resveratrol and is associated with a resveratrol trimer [46].

During this period, three resveratrol trimers, a pair of stereoisomers *trans*-suffruticosol D (**60**) and *cis*-suffruticosol D (**61**), and *cis*-gnetin H (**62**), were isolated from the seeds of *Paeonia Suffruticosa* (Paeoniaceae) [47]. The new resveratrol trimers all shared a common carbon skeleton, and the resveratrol units were related to the benzofuran rings.

From the plants of *Gnetum macrostachyum* (Gnetaceae) mentioned above, in addition to two more dimers, Sri-in et al. isolated a novel resveratrol trimer, macrostachyol B (**63**), which contains a dihydrofuran ring and an interesting bicyclic internal ring system created by the carbon bridge [36]. A new resveratrol trimer, gnetubrunol A (**64**), is probably related to a resveratrol trimer coupling with two dihydrobenzofuran rings [48].

Liu and co-workers studied the methanolic extract of roots of *Rheum lhasaense*, and isolated two new resveratrol trimers, rheumlhasol A and B (**65**, **66**). These two trimers are isomeride. The biosynthetic pathway of rheumlhasol B (**66**) is the gnetin C oxidative coupling with another resveratrol monomer. This is the first time resveratrol trimers were discovered in the plants of *Rheum* [49].

The discovery of the stilbene oligomers in the family Gramineae has been reported. Two new stilbene trimers, cystibenetrimerol A (**67**) and cystibenetrimerol B (**68**), were isolated from the EtOAc extract of the dried grass of *Cynodon dactylon* (L.) Pers. by successive chromatographic procedures (silica gel, Sephadex LH-20, MCI gel CHP 20P, and semi-preparative high performance liquid chromatography (HPLC)). The isolation and structures of two new stilbene trimers suggest that the ordinary grass from the *Poaceae* family might be another rich source of stilbene oligomers [50].

### 2.4. Resveratrol Tetramers

The majority of resveratrol tetramers contain a benzofuran moiety because the tetramers are primarily “dimers of dimers” [13]. Since 2010, 13 resveratrol tetramers (**69**–**81**) were isolated and identified, whereas, ten tetramers were discovered in the plants of Dipterocarpaceae (Table A1; Figure 5).

In Dipterocarpaceae plants, two new resveratrol tetramers, vatalbinoside A and B (**69**, **70**), share two common trans-oriented dihydrobenzofuran ring structures and a sequence of four -CH- groups [22]. Additionally, vatalbinoside B (**70**), a second instance of a *C*-glucopyranoside resveratrol tetramer, is the first where a *C*-glucopyranoside was isolated from the genus *Vatica* [22]. A dimeric dimer, vaticanol L (**71**), has a unique skeleton without a heterocyclic ring [51]. A novel resveratrol dimer, vateriaphenol F (**72**), with a unique C2-symetric structure and a new *O*-glucoside of REVs, vateriosides B (**73**), was isolated from *Vateria indica* [25]. In the heartwood of *Neobalanocarpus heimii*, Bayach et al. isolated three new resveratrol tetramers, heimiols C–E (**74**–**76**), all of which have two dihydrofuran rings, and heimiols D (**75**) is an oxidative tetramer of resveratrol [30]. The compounds malaysianol B and C (**77**, **78**) were isolated from the stem bark of *Dryobalanops lanceolate*. Malaysianol B (**77**) has a condensation type, initiated from the oxidative coupling reaction of two *ε*-viniferin molecules [52], and malaysianol C (**78**) is a novel symmetrical resveratrol tetramer, containing a tetrahydrofuran ring moiety and a unique tetrahydrofuran ring. Further research showed that the biosynthetic origin of malaysianol C (**78**) is from the condensation of two molecules of ε-viniferin as the precursor, and one of them will act as an epoxide derivative [53].

In the genus *Gnetum* (Gnetaceae), a resveratrol tetramer macrostachyol A (**79**), was condensed by oxidative coupling of a trimer, latifolol and, a resveratrol unit [36].

The last two resveratrol tetramers, cajyphenol A and cajyphenol B (**80**, **81**), were isolated from the stems of *Cayratia japonica* (Vitaceae) and contained a common carbon skeleton without a heterocyclic ring [54].

### 2.5. Resveratrol Pentamers

Only four resveratrol pentamers (**82**–**85**) were isolated from 2010 to 2017, and all the compounds were isolated from *Dipterocarpaceae* plants (Table A1; Figure 6). The first is hopeaside F (**82**), a new resveratrol pentamer discovered from the stem of the *Hopea utilis*, and is the third example of a *C*-glucopyranosyl resveratrol pentamer found in natural plants [42]. Two resveratrol pentamers, hopeasides A and B (**83**, **84**), were also isolated from the stem of *H. parviflora*. Both have the same carbon skeleton and contain the same novel part as hopeasides C and D (**55**, **20**) [29]. Upunoside E (**85**), a new *O*-glucoside of resveratrol pentamer, was purified as a pale yellow solid from an acetone-soluble extract of the leaves of *Upuna borneensis* (Dipterocarpaceae) by column chromatography [32].

### 2.6. Resveratrol Hexamers

During this period, six resveratrol hexamers (**86**–**91**) were separated and identified. Five of them were isolated from *Dipterocarpaceae* plants and another was identified from *Vitaceae* (Table A1; Figure 7).

Albiraminols A (**86**), condensed by a tetrameric resveratrol (vaticanol B) and a dimeric resveratrol, was isolated from the stem of *Vatica albiramis*, and is the first example bearing the blocking unit of vaticanol B [22]. Four resveratrol hexamers of vatcaside M (**87**) and vatcasides E–G (**88**–**90**) were isolated from three species, *Vatica bantamensis*, *V. chinensis*, and *V. albiramis*. Vatcasides E (**88**) and F (**89**) are two *O*-glucosides of (**87**). Vatcasides G (**90**) is an epimer of (**88**). They all contain a common partial structure of (−)-vaticanol B with a 3-(3,5-dihydroxyphenyl)-4,6-dihydroxy-2-(4-hydroxyphenyl)-2,3-dihydro-1*H*-inden-1-yl (4-hydroxyphenyl)methyl group [55].

The final new resveratrol hexamer, viniphenol A (**91**), was isolated from the vine stalks of *Vitis vinifera*. It contains three 2,3-*trans* configuration dihydrobenzofuran rings [56].

### 2.7. Resveratrol Heptamers

No new resveratrol heptamer was isolated from 2010 to 2017. Only two heptamers, pauciflorol D [57] and vaticanol J [58], have been reported from the stern bark of *Vatica pauciflora* and *V. rassak* in 2001 and 2004, respectively.

### 2.8. Resveratrol Octamers

A resveratrol octamer is the largest degree of polymerization of any resveratrol oligomer isolated to date (Table A1; Figure 8). Upunaphenol Q (**92**), the only instance of a resveratrol octamer in this period, was identified from the leaves of *Upuna borneensis* Sym, coupled with the dimeric structure of (−)-vaticanol B. It is the second instance of a resveratrol octamer [59]. Before this, only vateriaphenol A was isolated from the acetone extract of the stem bark of *Vateria indica* (Dipterocarpaceae) [60].

## 3. Pharmacological Activities

REVs have garnered interest due to their versatile bioactivities, including antimicrobial [61,62], antioxidant [63], and anticancer [64] activies. However, researchers have focused more on other activities, such as potential for the treatment of Alzheimer’s and Parkinson’s diseases. In 2015, Keylor et al. summarized some bioactivities of REVs, including anticancer, antioxidant, and modulation of enzymes [13]. Here, we aimed to provide a more comprehensive review on the progress in pharmacological activities. Anti-microbial, anti-Alzheimer’s disease, cardiovascular protection, anti-Parkinson’s disease, anti-tumor activities and other bioactivities are summarized below, with the exception of those which have been mentioned in the 2015 article [13].

### 3.1. Anti-Microbial Activities

Resveratrol and its oligomers play an important role in protecting plants from fungal and bacterial invasion. An evaluation of the anti-bacterial activity of REVs which were isolated from the stem bark of *Dryobalanops lanceolate*, against three Gram-positive strains, *Staphylococcus epidermidis*, *S. aureusm* and *S. xylosus*, had been performed. Two resveratrol tetramers, upunaphenol D and flexuosol A, showed potent antibacterial properties with a minimum inhibitory concentration (MIC) value of 25/75, 50/100, and 25/75 μmol/L, respectively. The results suggest that the disruption of the double bond resonance in the free resveratrol may attribute to the lower flexuosol A activity [52]. The resveratrol trimer α-viniferin showed significant activity against *Staphylococcus aureus* and *Escherichia coli* and showed moderate activity against *Salmonella paratyphi* [65]. In another assay, the resveratrol trimer α-viniferin, the resveratrol dimers ε-viniferin, and johorenol A, inhibited the growth of two methicillin-resistant *Staphylococcus aureus* (MRSA), ATCC 33591, and a HUKM strain obtained and characterized from clinical samples of infected patients in the University Kebangsaan Malaysia Hospital, Kuala Lumpur. α-viniferin and *ε*-viniferin showed a potent antibacterial activity on both MRSA strains at MIC at 100 and 400 μg/mL, respectively, whereas johorenol A showed activity on ATCC 33591 and HUKM strain with a MIC value of 100 μg/mL and 200 μg/mL, respectively. Either α-viniferin or *ε*-viniferin, in combination with vancomycin, exhibited an additive effect (0.5 < fractional inhibitory concentration (FIC) ≤ 2.0) against both MRSA strains. Johorenol A, in combination with vancomycin, also showed an additive effect on HUKM strains, whereas it demonstrated a synergistic interaction with vancomycin in killing ATCC 33591 strains (FIC < 0.5) [66]. A resveratrol trimer, davidiol A was capable of inhibiting the growth of both *S. uberis* and *B. subtilis* [2]. The dimer of *ε*-viniferin had potent antibiofilm activity against the pathogenic *Escherichia coli* O157:H7, inhibiting biofilm formation of *Escherichia coli* O157:H7 by 98% at 10 μg/mL [67]. Suffruticosol A, suffruticosol B, and vitisin A had better antibiofilm activities than resveratrol. Vitisin A displayed the most significant inhibitory activities on *E. coli* O157:H7, inhibiting biofilm formation by more than 90% at 5 μg/mL. The mechanism of the inhibition on *E. coli* O157:H7 biofilm formation was related to the ability of inhibiting fimbriae production [68]. A stereoisomer of hemsleyanols C [69] and four resveratrol tetramers, vaticanol B, vaticaphenol A, vateriaphenol B, and hopeaphenol, isolated from the ethyl acetate extracts of the leaves of *Hopea acuminata*, were found to inhibit protein splicing mediated by the *Mycobacterium tuberculosis* RecA intein in a nonspecific manner. The IC_50_ values for the five compounds were 3.4, 1.0, 1.7, 2.7, and 1.6 μmol/L, respectively [70].

In addition to antibacterial properties, REVs have antiviral properties. Studies on anti-herpetic activity found that vaticanol E, pauciflorol B, hopeaphenol A, pauciflorol C, vaticaffinol, and hemsleyanol D demonstrated significant activity against HSV-1 infection with IC_50_ values of 12.1 ± 0.5, 16.7 ± 0.5, 17.3 ± 0.5, 24.1 ± 0.6, 17.9 ± 0.3, and 9.1 ± 0.5 μmol/L, respectively, and against HSV-2 with IC_50_ values of 4.5 ± 0.1, 3.2 ± 0.5, 3.7 ± 0.2, 3.3 ± 0.1, 3.2 ± 0.3, and 3.8 ± 0.2 μmol/L, respectively. The anti-herpetic activity of these compounds may be related to the ability to enhance the transient release of reactive oxygen species [71].

### 3.2. Anti-Alzheimer’s Disease (AD)

Alzheimer’s disease (AD) is a devastating neurodegenerative disorder characterized by impaired memory and cognition. One of the major pathological hallmarks of AD in the brain is senile plaques that are composed of heterozygous amyloid-β (Aβ) peptides. Evidence indicates that accumulation of Aβ peptides in vulnerable brain regions plays a central role in AD pathogenesis [72]. In an anti-AD study, vitisinol C, scirpusin A, and *ε*-viniferin glucoside demonstrated significant anti-aggregative activity to prevent Aβ fibril formation with an EC_50_ value of 5 ± 3 [46], 0.7 ± 0.3, and 0.2 ± 0.3 μmol/L [73], respectively. The trimer miyabenol C demonstrated an ability to reduce Aβ generation by inhibiting β-secretase activity, which is related to the reduction of Aβ and sAPPβ both in vitro and in vivo in an AD model mice [74]. Furthermore, the REVs (−)-7a,8a-*cis*-*ε*-viniferin, *trans*-*ε*-viniferin, *cis*-*ε*-viniferin, *trans*-resveratrol, vitisinol C, vitisinol E, gnetin H, suffruticosol A, and suffruticosol B significantly inhibited baculovirus-expressed beta-site APP-cleaving enzyme 1 (BACE-1) in a concentration-dependent manner. The IC_50_ values were 1.29, 1.85, 2.21, 11.9, 4.01, 19.8, 0.34, 5.99, and 0.88 μmol/L , respectively [35]. The new resveratrol hexamer, viniphenol A (**91**) displayed protective activities against Aβ-induced toxicity in PC12 cells in a concentration-dependent manner [56].

In addition, four REVs, vaticahainol B, vaticanol E, pauciflorol B, and vatdiospyroidol, showed significant activities against AChE, which is seen as a potential treatment target for AD, with IC_50_ values of 18.9 ± 1.7, 12.0 ± 1.4, 10.9 ± 1.2, and 7.3 ± 1.8 μmol/L, respectively. Of note, the IC_50_ of vatdiospyroidol is closer to the positive control (±)-huperzine A (IC_50_ = 1.7 ± 0.3 μmol/L), a clinical anti-Alzheimer drug [24]. Two REVs, dipterocarpols A (**18**) and hopeahainol A, showed moderate AChE inhibitory activity with IC_50_ values of 8.28 μmol/L and 11.28 μmol/L, respectively [31].

### 3.3. Anti-Parkinson’s Disease (PD)

Parkinson’s disease (PD) is the second-most encountered neurodegenerative disorder after Alzheimer’s disease [75]. The aggregation of α-synuclein is one of the key pathogenic events in PD. Three stilbenes, piceatannol, ampelopsin A, and isohopeaphenol, were tested in lipid vesicle permeabilization assays for potential protection against membrane damage induced by aggregated α-synuclein. The viability of PC12 cells was examined to assess the preventive effects of these stilbenes against α-synuclein-induced toxicity. Piceatannol, a resveratrol monomer, inhibited the formation of α-synuclein fibrils and was able to destabilize preformed filaments at 100 μmol/L. It seems to induce the formation of small soluble complexes, protecting membranes against α-synuclein-induced damage. Further research showed that piceatannol protected cells against α-synuclein-induced toxicity; however, the oligomers tested, ampelopsin A and hopeaphenol, were less active [76].

### 3.4. Antitumor Activity

The antitumor activity of natural REVs is well documented. A variety of REVs exhibited cytotoxicity against various tumor cell lines. Hopeaphenol, vaticanol B, hemsleyanol D, and (+)-*α*-viniferin showed a strong antimelanoma effect against SK-MEL-28 melanoma cells. Other than vaticanol B, the other oligomers can selectively arrest cell cycle at the G1 phase, resulting in apoptosis of cancer cells [77].

The oligostilbene isomers, *cis*- and *trans*-suffruticosol D isolated from seeds of *P. suffruticosa*, exhibited remarkable cytotoxicity against human cancer cell lines including A549 (lung), BT20 (breast), MCF-7 (breast), and U2OS (osteosarcoma). *Trans*-suffruticosol D appeared to be slightly more potent (IC_50_ values: 9.93–20.8 μmol/L) than *cis*-suffruticosol D (IC_50_ values: 13.42–46.79 μmol/L) in the cancer cell lines tested, whereas it showed significantly less toxicity on the normal human cell lines, HMEC (breast) and HPL1A (lung). A mechanistic study demonstrated that *cis*- and *trans*-suffruticosol D exerted their antitumor effects by provoking oxidative stress, stimulating apoptosis, decreasing the mitochondrial membrane potential, inhibiting cell motility, and blocking the NF-κB pathway in human lung cancer cells. These studies suggest that *cis*- and *trans*-suffruticosol D could be promising chemotherapeutic agents against cancer [78]. In addition, vaticanol C showed a moderate activity against human lung cancer A549 cells (IC_50_ = 11.83 mmol/L). The polarity and stereochemistry of REVs might influence their cytotoxicity [51].

### 3.5. Cardiovascular Protection

Vitisin B displayed significant inhibitory activity on the migration of vascular smooth muscle cells, directly inhibiting platelet-derived growth factor (PDGF) signaling and enhancing the cell adhesiveness in cultured vascular smooth muscle cells via actin cytoskeleton recombination and phosphorylated tyrosine protein repartition [79]. Moreover, amurensin G had activities to relax endothelium-intact aortic rings, promote endothelial nitric oxide synthase (eNOS) phosphorylation, and nitric oxide (NO) production, and exert an effect on ER-dependent AMPK/PI3K pathways. Amurensin G might be useful to prevent atherosclerosis [4].

### 3.6. Liver-Protective Effect

The resveratrol dimer *ε*-viniferin displayed significant activity to protect Chang liver cells from hydrogen peroxide (H_2_O_2_) damage. When treated with *ε*-viniferin at 50 μmol/L and 100 μmol/L, the percentage of liver cell viability changed from 78.3% to 106.9% and 111.0%, respectively. The strong antioxidant activity plays an important role in the capacity to protect liver cells [80]. In another study, (−)-hopeaphenol, (+)-isohopeaphenol and (+)-α-viniferin, at a dose of 100 or 200 mg/kg, p.o., exhibited hepatoprotective effects in liver injuries in mice, induced with d-galactosamine (d-galN)/lipopolysaccharide (LPS), by reducing LPS-induced macrophage activation and the sensitivity of hepatocytes to TNF-α [81].

The resveratrol tetramer vitisin B exhibited a strong inhibition of HCV replication with an EC_50_ value of 6 nM and showed remarkably low cytotoxicity (EC_50_ >10 μmol/L). The mechanisms of action of vitisin B were related to the potent inhibition of a HCV NS3 helicase with IC_50_ 3 nM [82].

### 3.7. Other Activities

Using Discovery Studio software, Ye and coworkers analyzed the interaction between REVs (dimer: ε-viniferin, trimer: amurensins D, tetramer: vitisin A) and Fos/Jun molecules. Using intracerebroventricular injection and the hot plate tests in mice, they concluded that a low degree polymerization of resveratrol could enhance the central analgesic effect, which is related to an increase of the active groups and rigid structure. Also, the molecular docking method can be applied in virtual screening of the analgesic activity of REVs [83].

In a study for natural fatty acid synthase (FAS) inhibitors, cajyphenol A (**80**) and cajyphenol B (**81**), along with quadrangularin A, pallidol, and resveratrol, demonstrated significant fast-binding inhibitory activity on FAS with IC_50_ values of 1.63 ± 0.02, 1.49 ± 0.03, 7.50 ± 0.01, 11.1 ± 0.01, and 10.2 ± 0.01 μmol/L, respectively [54].

Two REVs, *trans*-*ε*-viniferin and r2-viniferin, were found to inhibit the cystic fibrosis transmembrane conductance regulator (CFTR) through high throughput screening [15]. Both REVs blocked CFTR-mediated iodide influx with IC_50_ values of about 20 μmol/L.

Six REVs, MIP (**1**), arahypin-11 (**30**), and arahypin-12 (**31**), together with arachidin-1, arachidin-3, and SB-1 (compound structure of a new metalolite, isolated from peanut (*Arachis hypogaea*) kernel, bears prenylated benzenoid and but-2-enolide moieties) demonstrated anti-adipogenic activities and showed cytotoxic effects on 3T3-L1 adipocyte differentiation cells at a concentration range of 1–10 μmol/L. The difference in their monomeric units and relative stereoconfigurations might play an important role in anti-adipogenic and cytotoxic activities [20].

(−)-hopeaphenol, hemsleyanol D, (+)-α-viniferin, and (−)-balanocarpol demonstrated an inhibitory activity against plasma glucose elevation in sucrose-loaded rats at doses of 100–200 mg/kg, p.o. and the possible mechanism of action was the inhibitory activity of gastric emptying, α-glucosidase, and aldose reductase [84]. These REVs showed potent activity in preventing plasma triglyceride elevation at a dose of 200 mg/kg p.o. in mice, with IC_50_ values of 32.9, 26.5, 23.2, 46.3, and 340 μmol/L, respectively. In addition, (−)-hopeaphenol and (+)-isohopeaphenol showed great potent inhibitory activity of pancreatic lipase with IC_50_ values of 32.9 and 26.5 μmol/L, respectively [85].

In addition, Scirpusin A, a REV dimer, showed interleukin-1β expression inhibitory activities on lipopolysaccharide (LPS)-induced THP-1 (the human acute monocytic leukemia) cells, with an inhibition rate of 35.8% at a concentration of 50 µg/mL [86].

## 4. Conclusions

In the wide variety of REVs, dimers and trimers account for the majority. Resveratrol octamers have the largest molecular weight and the least proportion. Many REVs contain the dihydrobenzofuran(s), *O*-glucosylation unit, and may be condensed by oxidative coupling of monomers, dimers, or trimers, and so on. In the past decades, REVs were mainly found in nine plant families. REVs have now been found in seven additional plant families. Plant chemists had been focusing on the Dipterocarpaceae family, searching for active stilbenes prior to 2010 [10,87]. Similarly, from 2010 to 2017, most novel REVs were obtained from the Dipterocarpaceae family, and the *Vatica*, *Shorea*, and *Hopea* genus. Among them, *Vateria* plants are the richest source [87]. This indicates that the Dipterocarpaceae family is the biggest natural source for obtaining highly polymerized REVs, or to isolate lower polymerized REVs as a base for synthetic highly polymerized oligomers. Compared to early studies, many new studies have concentrated on anti-Alzheimer’s disease, anti-Parkinson’s disease, and cardiovascular protection, in addition to bioactivities, such as antimicrobial, antioxidant, and antitumor activities. However, similar to a few years ago, the study of the structure–activity relationships of REVs is still lacking. In addition, obtaining a large amount of the naturally occurring resveratrol oligomers is difficult, owing to their low content and complex structure in plants, severely hampering their biological evaluation and related mechanism of action exploration in vivo. These deficiencies have limited the progression of REVs in drug development. Though these bioactivities have been mostly investigated in vitro at present, many of these REVs have shown significant bioactivities. With the progress in chemical synthesis technology, our enriched understanding of their chemistry and biology, and novel and potent Rev compounds continuing to be discovered, several promising REVs could be lead compounds for candidate drug discovery, and further development could serve as chemotherapeutic agents for cancers and other intractable diseases in the near future.

## Supplementary Materials

Supplementary File 1

## Appendix A

**Table A1:** The novel resveratrol oligomers isolated from plants over the period from 2010 to present.

No.	Chemical Component	Source	Part of Plant	Ref.
**Resveratrol Monomer**				
**Moraceae**				
1	cudrastilbene	*Cudrania tricuspidata*	roots	[19]
**Leguminosae**				
2	3,5,3′-trihydroxy-4′-methoxy-5′-isopentenylstilbene	*Arachis hypogaea*	seeds	[20]
3	chiricanine B	*Arachis hypogaea*	seeds	[21]
4	arahypin-13	*Arachis hypogaea*	seeds	[21]
5	arahypin-14	*Arachis hypogaea*	seeds	[21]
6	arahypin-15	*Arachis hypogaea*	seeds	[21]
**Resveratrol Dimer**				
**Dipterocarpaceae**				
7	vatalbinoside C	*Vatica albiramis*	stems	[22]
8	vatalbinoside D	*Vatica albiramis*	stems	[22]
9	vatalbinoside E	*Vatica albiramis*	stems	[22]
10	albiraminols B	*Vatica albiramis*	stems	[23]
11	vatalbinoside F	*Vatica albiramis*	stems	[23]
12	vaticahainols A	*Vatica mangachapoi.*	branches and twigs	[24]
13	vaticahainols B	*Vatica mangachapoi.*	branches and twigs	[24]
14	vaticahainols C	*Vatica mangachapoi.*	branches and twigs	[24]
15	vateriosides A	*Vateria indica*	leaves	[25]
16	roxburghiol A	*Shorea roxburghii*	roots	[26]
17	acuminatol	*Shorea acuminata*	stem barks	[27]
18	dipterocarpols A	*Dipterocarpus alatus*	stemwood	[28]
19	dipterocarpols B	*Dipterocarpus alatus*	stemwood	[28]
20	upunosides F	*Upuna borneensis*	leaves	[29]
21	upunosides G	*Upuna borneensis*	leaves	[29]
22	cordifoloside A	*Shorea cordifolia*	leaves	[30]
23	cordifoloside B	*Shorea cordifolia*	leaves	[30]
24	hopeasides D	*Hopea parviflora*	stems	[31]
25	heimiol B	*Neobalanocarpus heimii*	heartwood	[32]
**Vitaceae**				
26	amurensin O	*Vitis amurensis*	roots	[33]
**Paeoniaceae**				
27	(−)-7α,8α-*cis*-*ε*-viniferin	*Paeonia lactiflora*	seeds	[34]
**Leguminosae**				
28	arahypin 6	*Arachis hypogaea*	seeds	[35]
29	arahypin 7	*Arachis hypogaea*	seeds	[35]
30	arahypin-11	*Arachis hypogaea*	seeds	[20]
31	arahypin-12	*Arachis hypogaea.*	seeds	[20]
**Gnetaceae**				
32	macrostachyols C	*Gnetum macrostachyum*	roots	[36]
33	macrostachyols D	*Gnetum macrostachyum*	roots	[36]
34	gnemontanins A	*Gnetum montanum*	caulis	[37]
35	gnemontanins B	*Gnetum montanum*	caules	[37]
36	gnemontanins C	*Gnetum montanum*	caules	[37]
37	gnemontanins D	*Gnetum montanum*	caules	[37]
38	gnemontanins E	*Gnetum montanum*	caules	[37]
39	gnemontanins F	*Gnetum montanum*	caules	[37]
40	gnemontanins G	*Gnetum montanum*	caules	[37]
41	(−)-gnetuhainin P	*Gnetum montanum*	caules	[37]
42	(−)-gnetuhainin I	*Gnetum montanum*	caules	[37]
**Cyperaceae**				
43	longusol A	*Cyperus longus*	whole plant	[38]
44	longusol B	*Cyperus longus*	whole plant	[38]
45	longusol C	*Cyperus longus*	whole plant	[38]
**Resveratrol Trimer**				
**Dipterocarpaceae**				
46	malaysianol A	*Dryobalanops aromatica*	stem barks	[40]
47	malaysianol D	*Dryobalanops beccarii*	stem barks	[41]
48	hopeaside E	*Hopea utilis*	stems	[42]
49	hopeasides C	*Hopea parviflora*	stems	[30]
50	hopeachinols E	*Hopea chinensis*	stem barks	[43]
51	hopeachinols F	*Hopea chinensis*	stem barks	[43]
52	hopeachinol G	*Hopea chinensis*	stem barks	[43]
53	hopeachinols H	*Hopea chinensis*	stem barks	[43]
54	hopeachinols I	*Hopea chinensis*	stem barks	[43]
55	dipterocarpols C	*Dipterocarpus alatus*	stem wood	[28]
56	dipterocarpols D	*Dipterocarpus alatus*	stem wood	[28]
**Vitaceae**				
57	wenchowenol	*Vitis wenchowensis*	roots and stems	[44]
58	quinquangularol	*Vitis quinquangularis*	roots and stems	[45]
59	(*Z*)-*cis*-miyabenol C	*Vitis vinifera*	grapevine shoot	[46]
**Paeoniaceae**				
60	*trans-* suffruticosol D	*Paeonia suffruticosa*	seeds	[47]
61	*cis*-suffruticosol D	*Paeonia suffruticosa*	seeds	[47]
62	*cis*-gnetin H	*Paeonia suffruticosa*	seeds	[47]
**Gnetaceae**				
63	macrostachyol B	*Gnetum macrostachyum*	roots	[36]
64	gnetubrunol A	*Gnetum brunonianum*	roots	[48]
**Polygonaceae**				
65	rheumlhasol A	*Rheum lhasaense*	roots	[49]
66	rheumlhasol B	*Rheum lhasaense*	roots	[49]
**Gramineae**				
67	cystibenetrimerol A	*Cynodon dactylon*	dried grass	[50]
68	cystibenetrimerol B	*Cynodon dactylon*	dried grass	
**Resveratrol Tetramer**				
**Dipterocarpaceae**				
69	vatalbinoside A	*Vatica albiramis*	stems	[22]
70	vatalbinoside B	*Vatica albiramis*	stems	[22]
71	vaticanol L	*Vatica chinensis*	stems	[51]
72	vateriaphenol F	*Vateria indica*	leaves	[25]
73	vateriosides B	*Vateria indica*	leaves	[25]
74	heimiols C	*Neobalanocarpus heimii*	heartwood	[30]
75	heimiols D	*Neobalanocarpus heimii*	heartwood	[30]
76	heimiols E	*Neobalanocarpus heimii*	heartwood	[30]
77	malaysianol B	*Dryobalanops lanceolata*	stem barks	[52]
78	malaysianol C	*Dryobalanops lanceolata*	stem barks	[53]
**Gnetaceae**				
79	macrostachyol A	*Gnetum macrostachyum.*	roots	[36]
**Vitaceae**				
80	cajyphenol A	*Cayratia japonica*	stems	[54]
81	cajyphenol B	*Cayratia japonica*	stems	[54]
**Resveratrol Pentamer**				
**Dipterocarpaceae**				
82	hopeaside F	*Hopea utilis*	stems	[42]
83	hopeasides A	*Hopea parviflora*	stems	[29]
84	hopeasides B	*Hopea parviflora*	stems	[29]
85	upunosides E	*Upuna borneensis*	leaves	[32]
**Resveratrol Hexamer**				
**Dipterocarpaceae**				
86	albiraminols A	*Vatica albiramis*	stems	[22]
87	vatcaside M	*Vatica bantamensis*;	leaveas	[55]
		*Vatica chinensis*;		
		*Vatica chinensis*		
88	vatcasides E	*Vatica bantamensis*,	leaveas	[55]
		*Vatica chinensis;*		
89	vatcasides F	*Vatica bantamensis*,	leaveas	[55]
		*Vatica chinensis*;		
90	vatcasides G	*Vatica bantamensis*,	leaveas	[55]
		*Vatica chinensis*;		
**Vitaceae**				
91	viniphenol A	*Vitis vinifera*	vine stalks	[56]
**Resveratrol Octamer**				
**Dipterocarpaceae**				
92	upunaphenol Q	*Upuna borneensis*	leaves	[59]
