# Supplementary material for: Update on Phytochemistry and Pharmacology of Naturally Occurring Resveratrol Oligomers
Source: Molecules. 2017 Nov 24;22(12):2050. doi: 10.3390/molecules22122050 (PMC6149893; doi:10.3390/molecules22122050)
Supplement: Supplementary File 1 [file molecules-22-02050-s001.zip › Supplementary Files/table.docx]

**Table 1 The novel resveratrol oligomers isolated from plant over the period from 2010 to present**

| **No.** | | | | **Chemical component** | | | **Source** | **Part of plant** | **Ref.** |
| --- | --- | --- | --- | --- | --- | --- | --- | --- | --- |
| **Resveratrol monomer** | | | | | | |  |  |  |
|  | | | | **Leguminosae** | | | |  |  |
|  | | 1 | | 3,5,3′-trihydroxy-4′-methoxy-5′-isopentenylstilbene | | *Arachis hypogaea* | | seeds | [19] |
|  | | 2 | | chiricanine B | | *Arachis hypogaea* | | seeds | [21] |
|  | | 3 | | arahypin-13 | | *Arachis hypogaea* | | seeds | [21] |
|  | | 4 | | arahypin-14 | | *Arachis hypogaea* | | seeds | [21] |
|  | | 5 | | arahypin-15 | | *Arachis hypogaea* | | seeds | [21] |
|  | | | | **Moraceae** | |  | |  |  |
|  | | | 6 | cudrastilbene | | *Cudrania tricuspidata* | | roots | [20] |
| **Resveratrol dimer** | | | | | |  | |  |  |
|  | | | | **Dipterocarpaceae** | | | |  |  |
|  | | 7 | | vatalbinoside C | *Vatica albiramis* | | | stem | [22] |
|  | | 8 | | vatalbinoside D | *Vatica albiramis* | | | stem | [22] |
|  | | 9 | | vatalbinoside E | *Vatica albiramis* | | | stem | [22] |
|  | | 10 | | albiraminols B | *Vatica albiramis* | | | stem | [23] |
|  | | 11 | | vatalbinoside F | *Vatica albiramis* | | | stem | [23] |
|  | | 12 | | vaticahainols A | *Vatica mangachapoi.* | | | branches and twigs | [24] |
|  | | 13 | | vaticahainols B | *Vatica mangachapoi.* | | | branches and twigs | [24] |
|  | | 14 | | vaticahainols C | *Vatica mangachapoi.* | | | branches and twigs | [24] |
|  | | 15 | | vateriosides A | *Vateria indica* | | | leaves | [25] |
|  | | 16 | | roxburghiol A | *Shorea roxburghii* | | | roots | [27] |
|  | | 17 | | acuminatol | *Shorea acuminata* | | | stem bark | [28] |
|  | | 18 | | cordifoloside A | *Shorea cordifolia* Thwaites | | | leaves | [29] |
|  | | 19 | | cordifoloside B | *Shorea cordifolia* Thwaites | | | leaves | [29] |
|  | | 20 | | hopeasides D | *Hopea parviflora* | | | stem | [30] |
|  | | 21 | | heimiol B | *Neobalanocarpus heimii* | | | heartwood | [31] |
|  | | 22 | | dipterocarpols A | *Dipterocarpus alatus* | | | stem wood | [32] |
|  | | 23 | | dipterocarpols B | *Dipterocarpus alatus* | | | stem wood | [32] |
|  | | 24 | | upunosides F | *Upuna borneensis* | | | leaves | [33] |
|  | | 25 | | upunosides G | *Upuna borneensis* | | | leaves | [33] |
|  | | | | Vitaceae |  | | |  |  |
|  | | 26 | | amurensin O | *Vitis amurensis* | | | roots | [34] |
|  | | | | **Paeoniaceae** |  | | |  |  |
|  | | 27 | | (−)-7a,8a-cis-ε-viniferin | *Paeonia lactiflora* | | | seed | [35] |
|  | | | | **Leguminosae** |  | | |  |  |
|  | | 28 | | arahypin 6 | *Arachis hypogaea* | | | seed | [36] |
|  | | 29 | | arahypin 7 | *Arachis hypogaea* | | | seed | [36] |
|  | | 30 | | arahypin-11 | *Arachis hypogaea* | | | seed | [19] |
|  | | 31 | | arahypin-12 | *Arachis hypogaea.* | | | seed | [19] |
|  | | | | **Gnetaceae** |  | | |  |  |
|  | | 32 | | macrostachyols C | *Gnetum macrostachyum.* | | | roots | [37] |
|  | | 33 | | macrostachyols D | *Gnetum macrostachyum.* | | | roots | [37] |
|  | | 34 | | gnemontanins A | *Gnetum montanum* Markgr. | | | caulis | [38] |
|  | | 35 | | gnemontanins B | *Gnetum montanum* Markgr. | | | caulis | [38] |
|  | | 36 | | gnemontanins C | *Gnetum montanum* Markgr. | | | caulis | [38] |
|  | | 37 | | gnemontanins D | *Gnetum montanum* Markgr. | | | caulis | [38] |
|  | | 38 | | gnemontanins E | *Gnetum montanum* Markgr. | | | caulis | [38] |
|  | | 39 | | gnemontanins F | *Gnetum montanum* Markgr. | | | caulis | [38] |
|  | | 40 | | gnemontanins G | *Gnetum montanum* Markgr. | | | caulis | [38] |
|  | | 41 | | gnemontanins P | *Gnetum montanum* Markgr. | | | caulis | [38] |
|  | | 42 | | gnemontanins I | *Gnetum montanum* Markgr. | | | caulis | [38] |
|  | | | | **Cyperaceae** |  | | |  |  |
|  | | 43 | | longusol A | *Cyperus longus* | | | whole plant | [39] |
|  | | 44 | | longusol B | *Cyperus longus* | | | whole plant | [39] |
|  | | 45 | | longusol C | *Cyperus longus* | | | whole plant | [39] |
| **Resveratrol trimer** | | | | |  | | |  |  |
|  | | | | **Dipterocarpaceae** |  | | |  |  |
|  | | 46 | | malaysianol A | *Dryobalanops aromatica* | | | stem bark | [41] |
|  | | 47 | | malaysianol D | *Dryobalanops beccarii* | | | stem bark | [51] |
|  | | 48 | | hopeaside E | *Hopea utilis* | | | stem | [42] |
|  | | 49 | | hopeasides C | *Hopea parviflora* | | | stem | [30] |
|  | | 50 | | hopeachinolsE | *Hopea chinensis* | | | stem bark | [43] |
|  | | 51 | | hopeachinols F | *Hopea chinensis* | | | stem bark | [43] |
|  | | 52 | | hopeachinol G | *Hopea chinensis* | | | stem bark | [43] |
|  | | 53 | | hopeachinols H | *Hopea chinensis* | | | stem bark | [43] |
|  | | 54 | | hopeachinols I | *Hopea chinensis* | | | stem bark | [43] |
|  | | 55 | | dipterocarpols C | *Dipterocarpus alatus* | | | stem wood | [32] |
|  | | 56 | | dipterocarpols D | *Dipterocarpus alatus* | | | stem wood | [32] |
|  | | | | **Vitaceae** |  | | |  |  |
|  | | 57 | | wenchowenol | *Vitis wenchowensis* | | | roots and stems | [44] |
|  | | 58 | | quinquangularol | *Vitis quinquangularis* | | | roots and stems | [45] |
|  | | 59 | | (Z)-*cis*-miyabenolC | *Vitis vinifera* | | | grapevine shoot | [46] |
|  | | | | **Paeoniaceae** |  | | |  |  |
|  | | 60 | | *trans-* suffruticosol D | *Paeonia suffruticosa* | | | seed | [47] |
|  | | 61 | | *cis*-suffruticosol D | *Paeonia suffruticosa* | | | seed | [47] |
|  | | 62 | | *cis*-gnetin H | *Paeonia suffruticosa* | | | seed | [47] |
|  | | | | **Gnetaceae** |  | | |  |  |
|  | | 63 | | macrostachyol B | *Gnetum macrostachyum* | | | root | [37] |
|  | | 64 | | gnetubrunol A | *Gnetum brunonianum* | | | root | [48] |
|  | | | | **Polygonaceae** |  | | |  |  |
|  | | 65 | | rheumlhasol A | *Rheum lhasaense* | | | root | [49] |
|  | | 66 | | rheumlhasol B | *Rheum lhasaense* | | | root | [49] |
|  | |  | | **Gramineae** |  | | |  |  |
|  | | 67 | | cystibenetrimerol A | *Cynodon dactylon (L.)* Pers. | | | dried grass | [50] |
|  | | 68 | | cystibenetrimerol B | *Cynodon dactylon (L.) Pers.* | | | dried grass |  |
| **Resveratrol tetramer** | | | | |  | | |  |  |
|  | | | | **Dipterocarpaceae** |  | | |  |  |
|  | | 69 | | vatalbinoside A | *Vatica albiramis* | | | stem | [22] |
|  | | 70 | | vatalbinoside B | *Vatica albiramis* | | | stem | [22] |
|  | | 71 | | vaticanol L | *Vatica chinensis* | | | stem | [51] |
|  | | 72 | | vateriaphenol F | *Vateria indica* | | | leaves | [25] |
|  | | 73 | | vateriosides B | *Vateria indica* | | | leaves | [25] |
|  | | 74 | | heimiols C | *Neobalanocarpus heimii* | | | heartwood | [31] |
|  | | 75 | | heimiols D | *Neobalanocarpus heimii* | | | heartwood | [31] |
|  | | 76 | | heimiols E | *Neobalanocarpus heimii* | | | heartwood | [31] |
|  | | 77 | | malaysianol B | *Dryobalanops lanceolata* | | | stem bark | [52] |
|  | | 78 | | malaysianol C | *Dryobalanops lanceolata* | | | stem bark | [53] |
|  | | | | *Gnetaceae* |  | | |  |  |
|  | | 79 | | macrostachyol A | *Gnetum macrostachyum.* | | | root | [37] |
|  | | | | **Vitaceae** |  | | |  |  |
|  | | 80 | | cajyphenol A | *Cayratia japonica* | | | stem | [54] |
|  | | 81 | | cajyphenol B | *Cayratia japonica* | | | stem | [54] |
|  | |  | |  |  | | |  |  |
| **Resveratrol pentamer** | | | | |  | | |  |  |
|  | | | | **Dipterocarpaceae** |  | | |  |  |
|  | | 82 | | hopeaside F | *Hopea utilis* | | | stem | [42] |
|  | | 83 | | hopeasides A | *Hopea parviflora* | | | stem | [30] |
|  | | 84 | | hopeasides B | *Hopea parviflora* | | | stem | [30] |
|  | | 85 | | Upunosides E | *Upuna borneensis* | | | leaves | [33] |
| **Resveratrol Hexamer** | | | | |  | | |  |  |
|  | | | | **Dipterocarpaceae** |  | | |  |  |
|  | 86 | | | albiraminols A | *Vatica albiramis.* | | | stem | [22] |
|  | 87-90 | | | vatcaside M,  vatcasides E, F, G | *Vatica bantamensis, Vatica chinensis Vatica albiramis* | | | leaveas; stem;  stem bark | [55] |
|  | | | | **vitaceae** |  | | |  |  |
|  | | 91 | | viniphenol A | *Vitis vinifera* | | | vine stalk | [56] |
| **Resveratrol octamer** | | | | |  | | |  |  |
|  | | | | **Dipterocarpaceae** |  | | |  |  |
|  | | 92 | | upunaphenol Q | *Upuna borneensis* Sym | | | leaves | [59] |
