# Supplementary material for: Update on Phytochemistry and Pharmacology of Naturally Occurring Resveratrol Oligomers
Source: Molecules. 2017 Nov 24;22(12):2050. doi: 10.3390/molecules22122050 (PMC6149893; doi:10.3390/molecules22122050)
Supplement: Supplementary File 1 [file molecules-22-02050-s001.zip › Supplementary Files/Figures.doc]

| **Chemical structures of Resveratrol Oligomers** | | | | |
| --- | --- | --- | --- | --- |
|  |  | | |  |
| 1 | 2 | | | 3 |
|  |  | | |  |
| 4 | 5 | | | 6 |
| Fig.2 The structure of resveratrol monomers | | | | |
|  |  | | |  |
| 7 | 8 | | | 9: R1=R2=Glc  11: R1=Glc, R2=H |
|  |  | | |  |
| 10 | 12 | | | 13 |
|  |  | | |  |
| 14 | 15 | | | 16 |
|  |  | | |  |
| 17 | 18 | | | 19 |
|  |  | | |  |
| 20 | 21 | | | 22 |
|  |  | | |  |
| 23 | 24: 8b, 7b=*trans*  25: 8b, 7b=*cis* | | | 26 |
|  | | |  | |
| 27 | | | 28: R=H  29: R=OH | |
|  | | |  | |
| 30 | | | 31 | |
|  | | |  | |
| 32 | | | 33 | |
|  | | |  | |
| 34: 7a-OH  35: 7b-OH | | | 36 | |
|  | | |  | |
| 37 | | | 38: R=H  39: R=Et | |
|  | | |  | |
| 40: 7’α, 8’β | | | 41 | |
|  | | |  | |
| 42 | | | 43 | |
|  | | |  | |
| 44 | | | 45 | |
| Fig.3 The structures of resveratrol dimers (7-45) | | | | |
|  | | |  | |
| 46 | | | 47 | |
|  | | |  | |
| 48 | | | 49 | |
|  | | |  | |
| 50: R=OCH_3_  51: R=H | | | 52: R=OCH_3_  53: R=H | |
|  | | |  | |
| 54 | | | 55 | |
|  | | |  | |
| 56 | | | 57 | |
|  | | |  | |
| 58 | | | 59 | |
|  | | |  | |
| 60: 7’ 8’= *trans*  61: 7’ 8’= *cis* | | | 62 | |
|  | | |  | |
| 63 | | | 64 | |
|  | | |  | |
| 65: 7a,8a=*cis*  66: 7a, 8a=*trans* | | | 67 | |
|  | | | | |
| 68 | | | | |
| Fig.4 The structures of resveratrol trimers (46-68) | | | | |
|  | | |  | |
| 69 | | | 70 | |
|  | | |  | |
| 71 | | | 72 | |
|  | | |  | |
| 73 | | | 74 | |
|  | | |  | |
| 75 | | | 76 | |
|  | | |  | |
| 77 | | | 78 | |
|  | | | | |
| 79 | | | | |
|  | |  | | |
| 80 | | 81 | | |
| Fig.5 The structures of resveratrol tetramers (69-81) | | | | |
|  | |  | | |
| 82 | | 83 | | |
|  | |  | | |
| 84 | | 85 | | |
| Fig.6 The structures of resveratrol pentamers (82-85) | | | | |
|  | |  | | |
| 86 | | 87: R_1_=H, R_2_=H  88: R_1_=Glc, R_2_=H  89: R_1_=Glc, R_2_=Glc  90: R_1_=Glc, R_2_=H | | |
|  | | | | |
| 91 | | | | |
| Fig.7 The structure of resveratrol hexamers (86-91) | | | | |
|  | | | | |
| Fig.8 The structure of resveratrol octamer (92) | | | | |
